# Microstructural assessment of the locus coeruleus‐entorhinal cortex pathway and association with ATN markers in patients with cognitive impairment

**DOI:** 10.1002/alz.093705

**Published:** 2025-01-09

**Authors:** Marco Aiello, Pasquale Borrelli, Carlo Cavaliere, Moira Marizzoni, Federica Ribaldi, Valentina Garibotto, Max Scheffler, Ileana O Jelescu, Jorge Jovicich, Marco Salvatore, Giovanni B. Frisoni, Michela Pievani

**Affiliations:** ^1^ IRCCS SYNLAB SDN, Naples Italy; ^2^ Biological Psychiatric Unit, IRCCS Istituto Centro San Giovanni di Dio Fatebenefratelli, Brescia Italy; ^3^ Geneva Memory Center, Department of Rehabilitation and Geriatrics, Geneva University Hospitals, Geneva Switzerland; ^4^ Centre for Biomedical Imaging (CIBM), Geneva Switzerland; ^5^ Division of Radiology, Geneva University Hospitals, Geneva Switzerland; ^6^ Department of Radiology, Lausanne University Hospital (CHUV), Lausanne Switzerland; ^7^ Center for Mind Brain Sciences, University of Trento, Trento Italy; ^8^ University of Geneva, Geneva Switzerland; ^9^ Laboratory of Alzheimer's Neuroimaging and Epidemiology ‐ LANE, IRCCS Istituto Centro San Giovanni di Dio Fatebenefratelli, Brescia Italy

## Abstract

**Background:**

This study investigated microstructural features of the locus coeruleus to entorhinal cortex pathway (LC‐EC) in relation to amyloid (A), tau (T), neurodegeneration (N) markers and cognitive impairment in memory clinic patients.

**Method:**

124 participants were recruited from the Geneva Memory Clinic (n=30 cognitively unimpaired – CU; n=80 MCI and n=14 dementia ‐ CI) and underwent clinical assessment, 3T MRI scan including diffusion weighted imaging, amyloid PET, and tau PET. Diffusivity indices (fractional anisotropy ‐ FA, mean, axial and radial diffusivities ‐ MD, AxD, RD) were assessed in the LC‐EC pathway using a probabilistic atlas. A, T, N markers were assessed both as continuous and dichotomous measures. Differences in LC‐EC microstructure according to ATN markers and diagnosis were assessed with ANOVA models (FDR correction). Linear regression models were used to test whether LC‐EC pathway microstructure predicted cognitive impairment independently of ATN markers.

**Result:**

Lower FA (p=0.020) and higher MD, RD and AxD (p<0.005) was observed in participants with tau positivity in the EC (TEC+, Braak stage >1) compared to tau negative subjects (TEC‐). Higher MD, RD and AxD was observed in neurodegeneration positive (N+, medial temporal atrophy) versus negative (N‐) participants (p<0.001), and CI versus CU (p<0.016). There was no difference in LC‐TE microstructure between amyloid positive (A+) and negative (A‐) subjects (p>0.05) nor between tau positive (T+; Braak stage >4) and negative (T‐) subjects (p>0.05). The regression model showed that RD of the LC‐EC tract was associated with clinical diagnosis and mini mental state examination score independently of ATN markers (p<0.05).

**Conclusion:**

Our results indicate that LC‐EC microstructural measures, specifically RD, are sensitive in detecting CI and provide complementary information over ATN biomarkers. Associations with T suggest that LC‐TE microstructural alterations show regional specificity in the EC.

